# Arthrocentesis and Temporomandibular Joint Disorders: Clinical and Radiological Results of a Prospective Study

**DOI:** 10.1155/2013/790648

**Published:** 2013-11-11

**Authors:** Giacomo De Riu, Mirella Stimolo, Silvio Mario Meloni, Damiano Soma, Milena Pisano, Salvatore Sembronio, Antonio Tullio

**Affiliations:** ^1^UOC Maxillo-Facciale, Dipartimento di Scienze Chirurgiche, Microchirurgiche e Mediche, Università degli Studi di Sassari, V. le San Pietro 43, 07100 Sassari, Italy; ^2^Maxillofacial Unit, S. Maria degli Angeli Hospital, Pordenone, Italy

## Abstract

*Purpose*. We evaluated the efficacy of arthrocentesis in the treatment of temporomandibular joint (TMJ) disorders. *Material and Methods*. In this prospective clinical case series, 30 consecutive patients with TMJ disorders underwent arthrocentesis using saline and sodium hyaluronate injections. Outcome measures were TMJ pain, maximum mouth opening (MMO), joint noises, and anatomical changes in the TMJ architecture. Patients were evaluated using cone-beam computed tomography (CBCT) and magnetic resonance imaging (MRI) at the beginning of treatment and 60 days after the last arthrocentesis. Pretreatment and posttreatment clinical parameters were compared using paired and unpaired *t*-tests, and McNemar's test was used to evaluate CBCT and MRI changes (*P* < 0.05). *Results*. At 1-year follow-up examinations, visual analogue scale scores indicated that pain was reduced significantly and mean postoperative MMO was increased significantly. CBCT findings showed no significant change, and MRI showed only slight reductions in inflammatory signs. *Conclusions*. Within the limitations of this study, we can conclude that arthrocentesis is a simple, minimally invasive procedure with a relatively low risk of complications and significant clinical benefits in patients with TMJ disorders. This trial is registered with NCT01903512.

## 1. Introduction

 Temporomandibular disorders (TMDs) represent a wide range of functional changes and pathological conditions affecting the temporomandibular joint (TMJ), masticatory muscles, and other components of the oromaxillofacial region. In recent years, TMD has become a frequent cause for seeking medical assistance. The number of patients with TMDs is increasing, probably due to psychological tension in modern society [1]. According to well-accepted psychophysiological concepts, occlusal problems and emotional stress are the most serious aetiological factors [2–7]. However, the causes of TMD are far more complex. A comprehensive understanding requires consideration of the whole masticatory apparatus and the intra-articular situation.

Due to the largely nonspecific nature of initial problems, patients are not typically referred to specialists until symptoms have evolved and, in many cases, after irreversible morphological and functional changes have occurred. Characteristic symptoms of TMJ disorders include pain, changes in mandibular mobility (reduced mouth opening (hypomobility) or, in contrast, hypermobility and luxation), clicking, and grinding.

 TMJ disorders may be treated conservatively or surgically. Conservative treatments include the use of bite wafers, rehabilitation exercises, isometric exercises, masticatory muscle massage, analgesic treatment, thermotherapy, and laser therapy. Surgical treatments can be invasive (open approaches) or minimally invasive, including arthrocentesis and arthroscopy [8].

 New insights into the joint pathology of internal derangements have been provided by observations made during TMJ arthroscopic lysis and lavage and analysis of the outcomes of such treatments. The physical actions of lysis and lavage in the superior joint space, which reduce inflammation rather than repositioning the disc, are believed to be responsible for the success of arthroscopic surgery [9, 10]. This finding has increased the use of TMJ arthrocentesis procedures to obtain symptomatic relief and restore the normal range of motion [9] and has made more aggressive approaches, such as disc replacement or repair, condylar shaving, and high condylectomy, less common.

 In this study, we sought to analyse the clinical results and eventual anatomical changes induced by arthrocentesis in the treatment of internal derangement of the TMJ. To our knowledge, this study is the first to evaluate the clinical outcomes of arthrocentesis by comparing pre- and posttreatment morphological parameters using cone-beam computed tomography (CBCT) and magnetic resonance imaging (MRI).

## 2. Materials and Methods

### 2.1. Study Design

In this prospective clinical study, clinical and imaging data from 30 consecutive patients treated with TMJ arthrocentesis and sodium hyaluronate (SH) injection were analysed. The investigation was conducted according to the principles of the 1975 Helsinki Declaration for biomedical research involving human subjects, as revised in 2004, and was approved by the Research Committee of the Department of Surgical, Microsurgical, and Medical Science, University of Sassari. At preliminary visits, all patients were duly informed of the nature of the study.

### 2.2. Selection Criteria

Patients of any ethnic group and gender who were aged ≥18 years, in good general health, and physically and psychologically able to undergo TMJ arthrocentesis were included in the study.

Inclusion criteria were TMJ pain at rest or evoked by palpation, mandibular distraction or forced opening manoeuvres, mandibular hypomobility, TMJ noises or clicking, failure of conservative treatments alone (nonsteroidal anti-inflammatory drugs (NSAIDs) and gnathological treatment), and concomitant occlusal bite therapy. Exclusion criteria were degenerative joint diseases, such as osteoarthritis, rheumatoid arthritis, and gout; history of condylar fractures or TMJ trauma; previous TMJ surgery; and poor compliance. According to these criteria, 30 patients treated with TMJ arthrocentesis were selected for this study.

### 2.3. Preoperative Evaluation

Patients' informed consent was obtained before clinical examinations were conducted and medical histories were collected. Patients' records were reviewed retrospectively to collect data on demographic characteristics, histories, preoperative physical examination findings (pain, joint noises, and dysfunction), and the results of MRI and CBCT analyses. Two major categories of TMJ disorder were encountered: anterior disc displacement (AD; *n* = 26 patients, group A) and other less common disorders, such as osteophytes, signs of soft-tissue inflammation, and TMJ structural alterations (*n* = 4, group B).

MRI was performed using the following parameters: 3 mm section thickness, 320 × 224 matrix, and 120 × 120 mm field of view. MR images were corrected horizontally with respect to the long axis of the condyle. Sequential bilateral oblique sagittal images were acquired with the subject's mouth closed and at the maximum open-mouth position.

An independent radiologist blinded to the patients' data assessed MRI data using established criteria. Joint status was first assessed by determining whether the disc was positioned normally, defined as the superior location (12 o'clock position) of the posterior band of the disc relative to the condyle, or whether AD was present. Deformity was then assessed by evaluating biconcave disc morphology, enlargement of the posterior band, and thickness. Disc dynamics were categorised as mobile or immobile (fixed or “stuck” in closed and open positions). Osteoarthrosis (OA) was defined by the presence of condylar deformities associated with flattening, subchondral sclerosis, surface irregularities, erosion, and osteophytes. The presence of joint effusion (JE) was evaluated. On T2-weighted images, JE was identified as an area of high signal intensity in the region of the joint space. Bone marrow oedema (BMO) was defined by the presence of a hypointense signal on T1-weighted images and a hyperintense signal on T2-weighted images.

**Figure 1:**
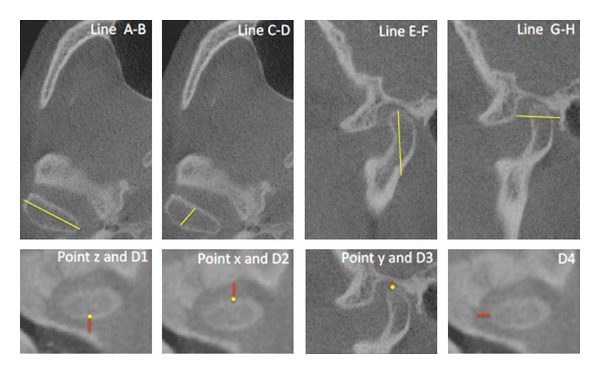
CBCT landmarks.

Morphometric analysis of the mandibular condyle and glenoid cavity was performed using CBCT images. All abnormalities, such as the presence of osteophytes, geodesic erosion of the cavity, and morphological changes, were recorded. Linear and angular measurements were made to evaluate the mediolateral orientation of the condyle on axial views and the positions and orientations of the mandibular condyles with respect to the cavity walls on sagittal and coronal views (Figure 1). The following landmarks and reference planes were involved:

- point x: on a sagittal view, the most prominent point of the outline of the ventral condyle corresponding to the insertion of the anterior joint capsule;
- point y: the most cranial point on the cranial contour of the condyle;
- point z: the dorsal condylar projection of point x in the horizontal plane;
- condylar angle: formed between the major mediolateral axis of the condyle and the axis drawn between the most projecting points of the dorsal surfaces of the two mandibular condyles (this measurement defined the orientation of the condyle in the horizontal plane);
- line AB: the maximum mediolateral width of the condyle;
- line CD: the maximum dorsoventral width of the condyle;
- line EF: the maximum craniocaudal length of the condyle;
- line GH: the maximum dorsoventral length of the glenoid fossa;
- D1: distance between the condyle (point z) and the temporal bone;
- D2: distance between the condyle (point x) and the articular eminence;
- D3: distance between the condyle (point y) and the posterior wall of the glenoid fossa;
- D4: distance between the condyle and the medial wall of the glenoid fossa.

All CBCT measurements were taken before treatment and repeated 60 days after-treatment.

**Figure 2:**
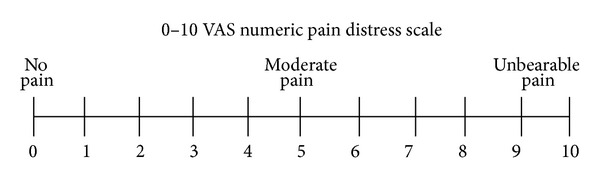
VAS.

 The following parameters were also evaluated:

- pain, measured with a visual analogue scale (VAS; Figure 2);
- maximum mouth opening (MMO), measured between the incisal edges of the maxillary and mandibular incisors;
- presence or absence of articular noises.

At follow-up visits, patients were also invited to complete an original satisfaction form, assessing treatment effectiveness (benefit to the patient) and tolerability on scales ranging from 0 to 4 (0, poor; 1, mild; 2, moderate; 3, good; 4, very good). 

Pretreatment and posttreatment MMO and levels of pain and dysfunction were compared using paired and unpaired *t*-tests, and McNemar's test was used to evaluate MRI and CBCT changes (*P* < 0.05).

### 2.4. Clinical Procedure

**Figure 3:**
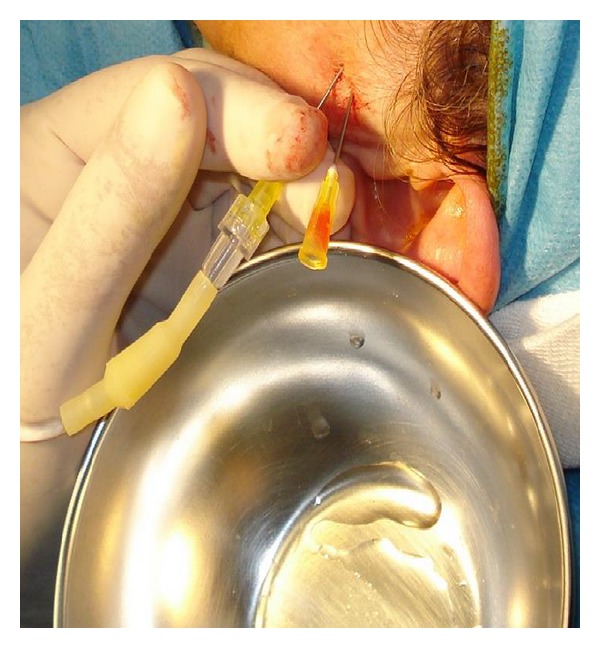
Clinical procedure: with the mouth widely open and the mandible in protruded position, introduction of the needles and injection of saline to fill the joint space and establish the flow of the NaCl solution are done.

Arthrocentesis of the superior joint space was performed under aseptic conditions and local anaesthesia. The patient was seated at a 45° angle, and the sites of needle insertion were marked on the skin according to the method suggested by Nitzan et al. [11]. A line was drawn from the middle of the tragus to the outer canthus. Entry points were marked along this canthotragal line. The first point, corresponding to the glenoid fossa, was marked 10 mm from the midtragus and 2 mm below the canthotragal line. A second point, corresponding to the articular eminence, was marked 10 mm from the first point and 10 mm below the line. Then, 2 mL articaine chlorhydrate with 1 : 100,000 adrenaline (Pierrel S.p.A., Milan, Italy) was injected to block the articular branch of the auriculotemporal nerve. The patient was asked to open the mouth wide, and the mandible was held in the protruded position. A 19-gauge needle was then introduced at the first point, and 2–4 mL NaCl solution (0.9% saline) was injected to fill the joint space. Another 19-gauge needle was then inserted at the second point to establish flow of the solution through the joint space. Saline was then injected under pressure through the first needle into the superior joint space; the second needle provided the outflow. In total, 300–400 mL NaCl solution (0.9% saline) was used for lavage (Figure 3). Finally, 2 mL SH (Hyalgan, Fidia, Italy) was injected into the superior joint space before removal of the needles. The patient's mandible was gently manipulated in the vertical, protrusive, and lateral directions to free up the disc. The use of NSAIDs and muscle relaxants for 1 week was advised. We did not administer pre- or posttreatment antibiotics.

### 2.5. Outcome Measures

- MRI: presence of disc interference, change in the position or conditions of the articular disc, and change in the relationship with the mandibular condyle.
- CBCT: morphometric changes in the mandibular condyle and glenoid cavity.
- Disappearance or significant reduction in pain, assessed using a VAS.
- Change in MMO.
- Disappearance, reduction, or increase in articular noise.

Pre- and posttreatment clinical parameters were compared using paired and unpaired *t*-tests, and McNemar's test was used to evaluate CBCT and MRI changes.

## 3. Results

In total, 30 consecutive patients (24 women, 6 men; age, 25–62 years) with TMJ disorders for which conservative management had failed were selected for this study. Disc displacement was observed in 26 patients, and only four patients presented other TMJ disorders (osteophytes, signs of soft-tissue inflammation, and TMJ structural alterations).

### 3.1. Pain

All patients had moderate to severe pain, with preoperative VAS scores ranging from 6 to 10 (mean, 8.26 ± 0.88). Pain decreased significantly (*P* < 0.001) during the long-term follow-up period, with posttreatment VAS scores ranging from 0 to 9 (mean, 2.03 ± 2.80). Both groups also showed significant reductions in pain (*P* < 0.001) when analysed individually.

VAS scores decreased significantly (*P* < 0.001) in the group with AD from a preoperative mean of 8.45 ± 0.75 (range, 7–10) to a long-term postoperative mean of 1.77 ± 2.49 (range, 0–9). In the group with other disorders, preoperative VAS scores ranged from 6 to 8 (mean, 7.25 ± 0.95) preoperatively and from 0 to 9 (mean, 3.5 ± 4.35) at the final follow-up evaluation (Table 1).

### 3.2. MMO

Table 1 summarises the findings of preoperative, immediately postoperative, and final clinical examinations. All patients experienced a significant increase in MMO (*P* < 0.001) immediately after arthrocentesis. Preoperative MMO values ranged from 20 to 40 mm (mean, 25.3 ± 5.5 mm), whereas postoperative MMO values ranged from 30 to 55 mm (mean, 43.8 ± 5.6 mm). At 1-year follow-up evaluations, MMO values ranged from 15 to 50 mm (mean, 37.1 ± 8.4 mm), representing a significant increase from pretreatment values (*P* < 0.001).

In patients with AD, immediate postoperative and long-term postoperative MMO values were significantly higher than prearthrocentesis values (*P* < 0.001). MMO values ranged from 20 to 40 mm (mean, 25.3 ± 5.7 mm) before arthrocentesis, from 30 to 55 mm (mean, 43.2 ± 5.5 mm) immediately after treatment, and from 25 to 50 mm (mean, 37.6 ± 6.3 mm) 1 year after treatment. In patients with other disorders, immediate postoperative and long-term postoperative MMO values were also significantly higher than pretreatment values (*P* < 0.001). Pretreatment MMO values ranged from 20 to 30 mm (mean, 25.2 ± 4.9 mm), immediate posttreatment values ranged from 40 to 53 mm (mean, 47.0 ± 5.7 mm), and long-term posttreatment values ranged from 15 to 45 mm (mean, 34.5 ± 13.3 mm; Table 2).

### 3.3. TMJ Noises

Before arthrocentesis, 53.8% of patients had clinically detectable joint noises; this proportion increased to 76.9% immediately after the procedure. Joint noises increased from 45% to 72% in patients with AD (group A) but were not affected by arthrocentesis in patients with other disorders.

### 3.4. MRI

**Figure 4:**
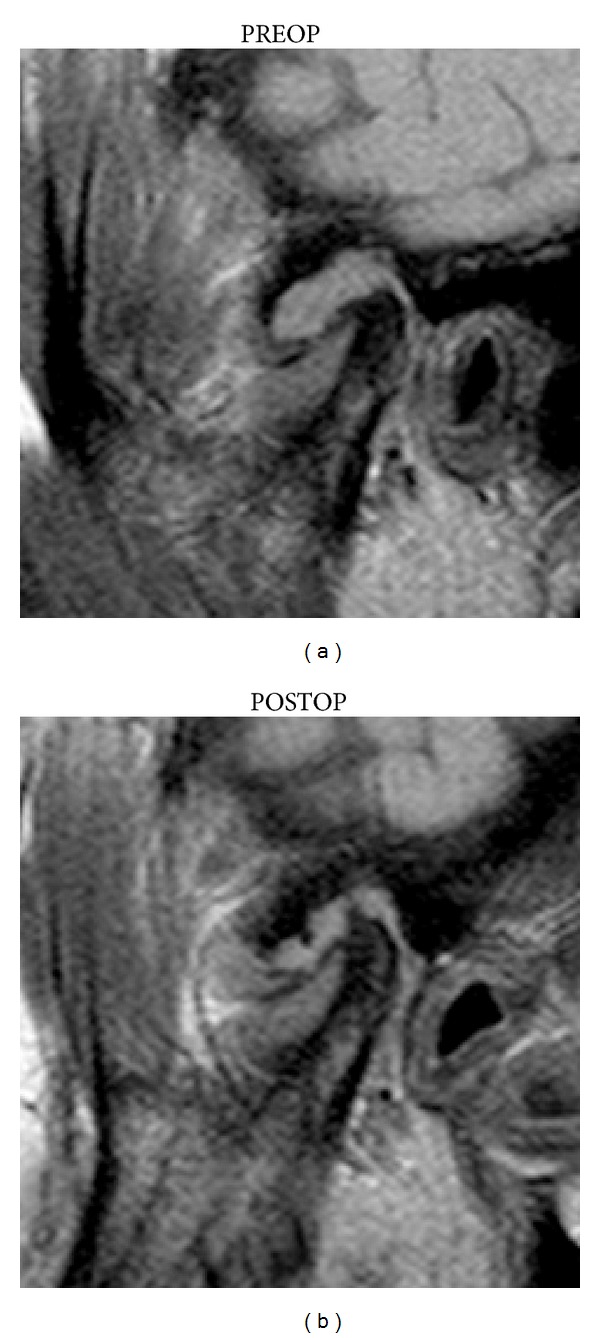
MRI: JE was identified as a high signal intensity area in the region of the joint space; note the mild reduction of inflammatory signs.

In the 26 patients with AD, no change in displacement or disc morphology was detected on MR images after treatment. Two of eight JE cases persisted after treatment (*P* = 0.15) (Figure 4).

BMO was present in eight joints and did not disappear in any case (Figure 4). OA did not change after treatment (Table 3).

### 3.5. CBCT

Posttreatment CBCT images showed no significant change in the TMJ bony structure (Table 4). No complication was detected intra- or postoperatively.

Patients' subjective scoring of treatment effectiveness yielded an average value of 3.45, corresponding to a good degree of satisfaction. The tolerability of arthrocentesis was between “moderate” and “good”, with an average score of 2.45.

## 4. Discussion

 Inflammatory and noninflammatory TMJ diseases are typically associated with structural alterations in joint tissues, such as cartilage degradation and subchondral bone alterations, which reflect the responses of cells, extracellular matrix macromolecules, collagen, and proteoglycans to articular load changes. In inflammatory TMJ diseases, various mediators—particularly cytokines—may be responsible for rearrangement of the extracellular matrix in joint tissues, altering normal cell reactions and allowing enzymatic degradation of the matrix. Collagenases and matrix metalloproteinases (MMPs), zinc-containing proteins with enzymatic activity, likely play roles in this process. Macromolecular degradation of the matrix determines physical and biological deterioration of the tissues and promotes the disease, because the degradation fragments, proteoglycans, and collagen released into the synovial fluid generate inflammatory pain, with further release of MMPs.

 Despite clinical evidence of disc displacement in TMJ internal derangement, current concepts suggest that a change in disc position is not a primary factor in TMJ pain or dysfunction. Instead, alterations in joint pressure (negative intra-articular pressure) and a variety of biochemical constituents of the synovial fluid (failure of lubrication) may lead to clicking and derangement of the TMJ [9, 12, 13].

 Thus, arthrocentesis may act by allowing the elimination of hyperviscous medium with catabolites and inflammatory cells, thereby counteracting the degeneration of tissues. Hyaluronic acid is the main component of the synovial fluid and cartilage matrix; it plays an important role in homeostasis of the TMJ due to its important viscoelastic properties, with a “bearing effect” against impact, and its analgesic effect. Hyaluronic acid can reduce the production of proinflammatory substances and vascular permeability and protect cell damage mediated by free radicals. Its application in different articulations has been reported. In addition to an immediate response (e.g., improved mastication ability), it also induces long-term modifications, as is typical of structure-modifying drugs. Hyaluronate thus has a slow symptomatic action, but is persistent, with a so-called “tail effect.” In regeneration induced in degenerated arthritic tissues with slow metabolism, injection of exogenous hyaluronic acid stimulates the production of endogenous hyaluronate by synoviocytes. The immediate action, however, is explained by a reduction in pain mediators when infiltrated into an inflamed joint with hypomobility and functional limitations.

 Degenerative joint disease is characterised by decreased concentration, molecular weight, and degree of polymerisation of endogenous hyaluronic acid, which involves reduced viscosity of the liquid, resulting in increased susceptibility to damage of the articular heads due to cartilage erosion mediated by exogenous phospholipases.

 Lavage of the upper joint space reduces pain by removing inflammatory mediators from the joint and increases mandibular mobility by removing intra-articular adhesions, eliminating the negative pressure within the joint, recovering disc and fossa space, and improving disc mobility, which reduces the mechanical obstruction caused by AD [13–18].

 Arthrocentesis has developed as a natural consequence of the success of arthroscopic lavage and lysis for the treatment of internal derangements [9]. Nitzan et al. [14] described arthrocentesis as the simplest form of surgery in the TMJ, seeking to release the articular disc and to remove adhesions between the disc surface and the mandibular fossa by means of hydraulic pressure from irrigation of the upper chamber of the TMJ [12, 14, 16, 25]. Studies to determine whether the effects of arthrocentesis on internal derangements are merely palliative or provide long-term relief of the associated symptoms have shown that arthrocentesis can produce long-term relief of pain and dysfunction in patients with internal derangements of the TMJ [19, 20].

 Arthrocentesis has been reported to be up to 91% effective in treating patients with AD without reduction [15, 21, 22]. In this study, 100% of patients showed significant reduction in pain after arthrocentesis. This pain reduction is attributed to high-pressure irrigation, which washes away inflammatory mediators, providing immediate pain relief. Failure of pain relief may be due to pain originating from causes other than internal derangement.

Arthrocentesis under sufficient pressure can also remove adhesions, widen joint spaces, and improve mouth opening [9, 23, 24]. In patients who presented with limited mouth opening, significant improvement was seen in the immediate postoperative period; along with a reduction in pain, mouth opening increased further from months 3 to 6. The mean increase in MMO was 23.6 mm. 

 In cases in which TMJ arthrocentesis fails to achieve the desired outcome, several factors should be considered. Appropriate case selection is important, because this technique seems to be ineffective in certain conditions, such as in cases with bony changes, fibroankylosis, and perforation of the disc [23, 25]. Even when these indications are evident, other associated factors, such as muscle spasm, must also be brought under control prior to arthrocentesis. When arthroscopy or open joint surgery is indicated, but the clinician is uncertain of the diagnosis, arthrocentesis may be used as a simple interim measure that can confirm the need for more invasive procedures [16, 26].

 Major disadvantages of arthrocentesis are the failure to directly show intra-articular pathology, the scarce possibility of pathological tissue biopsy, and the difficulty of treating more mature adhesions [27]. Sweeping and other nonoperative arthroscopic manoeuvres, which can be performed with arthroscopic lysis and lavage, are not possible with arthrocentesis alone. Transient facial paresis due to the local anaesthetic or swelling of the neighbouring tissues caused by perfusion of solution may occur during arthrocentesis [28].

Radiographic assessment of the temporomandibular joint using CBCT imaging allows clinicians to find out subtle osteoarthrosis alterations such as subchondral cysts and sclerosis, osteophyte formation, surface erosion, and bony remodeling. MRI is considered the gold standard for the study of intracapsular disorders. The MRI allows to evaluate the morphologic features of the disc and its location with respect to the condyle in both closed and open-mouth positions. Other MRI signs that can suggest TMJ dysfunction include thickening, perforations of retrodiscal layers, or joint effusion. MRI therefore allows to detect degenerative signs, invisible to CBCT, allowing early precise diagnosis of different TMJ disorders.

 Thereby comparative imaging studies can prevent possible evolution to more advanced and irreversible phases, characterized by osteoarthrosis changes.

## 5. Conclusions

 Arthrocentesis of the TMJ is a minimally invasive method of treatment, located at the boundary between conservative and surgical therapy. It is usually performed on an outpatient basis under local anaesthesia. It is used for acute closed or open lock caused by displacement of the articular disc and for the treatment of degenerative inflammatory joint disease. The main objectives of arthrocentesis are to wash out inflammatory mediators, release the disc, break up adhesions, eliminate pain, and improve joint mobility. It is a method with a minimum number of complications; it is simple and not demanding in terms of instruments, and it can be performed repeatedly. These features make arthrocentesis a valid treatment option for patients with low and mild TMJ disorders.

## Figures and Tables

**Table 1:** Comparison of pretreatment and posttreatment pain.

Period	Mean	Group A	Group B	*P*	*P* (group A)	*P* (group B)
Preoperative	8.26 ± 0.088	8.45 ± 0.75	7.25 ± 0.95	<0.001	<0.001	<0.001
1-year postoperative	2.03 ± 2.80	1.77 ± 2.49	3.5 ± 4.35			

**Table 2:** Comparison of pretreatment and posttreatment mouth opening.

Period	Mean	Group A	Group B	*P*	*P* (group A)	*P* (group B)
Preoperative	25.3 ± 5.5	25.3 ± 5.7	25.2 ± 4.9	<0.001	<0.001	<0.001
Postoperative	43.8 ± 5.6	43.2 ± 5.5	47.0 ± 5.7			
1 year	37.1 ± 8.4	37.6 ± 6.3	34.5 ± 13.3			

**Table 3:** Changes in MRI findings.

	Preoperative	Postoperative	*P *
Disc position			
No AD	4	4	
AD	26	26	

Disc mobility			
Normal	30	30	
Stuck			

Joint effusion			0.15
Absent	22	24	
Present	8	6	

Bone marrow oedema			
Absent	25	25	
Present	5	5	

Osteoarthrosis			
Absent	27	27	
Present	3	3	

AD: anterior displacement.

**Table 4:** Changes in CBCT findings.

	Preoperative	Postoperative
Condylar angle	19.39 ± 4.33	19.39 ± 4.33
A-B	17.36 ± 1.44	17.36 ± 1.44
C-D	7.95 ± 0.92	7.95 ± 0.92
E-F	3.53 ± 0.51	3.53 ± 0.51
G-H	13.56 ± 1.16	13.56 ± 1.16
D1	2.48 ± 0.66	2.48 ± 0.66
D2	3.09 ± 0.67	3.09 ± 0.67
D3	2.58 ± 0.62	2.58 ± 0.62
D4	4.28 ± 0.99	4.28 ± 0.99
